# Cannabinoids for the treatment of cannabis use disorder: New avenues for reaching and helping youth?

**DOI:** 10.1016/j.neubiorev.2021.11.033

**Published:** 2021-11-22

**Authors:** Kristine Rømer Thomsen, Birgitte Thylstrup, Emily A. Kenyon, Rachel Lees, Lone Baandrup, Sarah W. Feldstein Ewing, Tom P. Freeman

**Affiliations:** a Centre for Alcohol and Drug Research, Department of Psychology and Behavioral Sciences, Aarhus University, Denmark; b Department of Psychology, University of Rhode Island, USA; c Addiction and Mental Health Group (AIM), Department of Psychology, University of Bath, UK; d Mental Health Centre Copenhagen and Department of Clinical Medicine, University of Copenhagen, Denmark

**Keywords:** Cannabis use disorder, Cannabis, Treatment, Youth, Pharmacotherapy, Cannabinoids, Cannabidiol, Δ-9-Tetrahydrocannabinol, FAAH inhibitors

## Abstract

Cannabis use peaks during adolescence and emerging adulthood, and cannabis use disorder (CUD) is associated with a wide range of adverse outcomes. This is particularly pertinent in youth, because the developing brain may be more vulnerable to adverse effects of frequent cannabis use. Combining evidence-based psychosocial interventions with safe and effective pharmacotherapy is a potential avenue to improve youth outcomes, but we lack approved CUD pharmacotherapies. Here, we review new potential avenues for helping youth with CUD, with a particular focus on cannabinoid-based treatments. Evidence from placebo-controlled RCTs suggests synthetic delta-9-tetrahydrocannabinol (THC) decreases withdrawal symptoms, but not cannabis use, in adults with daily cannabis use/CUD, while findings regarding formulations containing THC combined with cannabidiol (CBD) are mixed. Preliminary evidence from two placebo-controlled RCTs in adults with CUD suggests that both Fatty Acid Amide Hydrolase inhibitors and CBD can reduce cannabis use. However, larger trials are needed to strengthen the evidence. Findings from adults point to cannabinoid-based treatments as a potential strategy that should be examined in youth with CUD.

## 1. Cannabis and changes in potency

Cannabis is the most frequently used drug globally, with nearly 4% using cannabis in the past year (United Nations Office on Drugs and Crime, 2020). Globally, about 13 million people are estimated to meet criteria for Cannabis Use Disorder (CUD: recurrent use of cannabis despite adverse consequences, causing clinically significant impairments or distress (American Psychiatric Association, 2013)) accounting for a global burden of disease of two million disability adjusted life years (GBD, 2018).

Cannabis contains at least 144 different cannabinoids, of which the main psychoactive component is Δ-9-tetrahydrocannabinol (THC, Hanus et al., 2016). THC produces its effects by acting on the endocannabinoid system, which includes cannabinoid receptors (e.g., CB1 and CB2), endocannabinoids that bind to these receptors (such as ananamide and 2-arachidonoylglycerol), and enzymes involved in their breakdown such as Fatty Acide Amide Hydrolase (FAAH). THC is a partial agonist at CB1 receptors, and frequent cannabis use results in downregulation of CB1 receptors with a potential to reverse with abstinence (D’Souza et al., 2016; Hirvonen et al., 2012). Another main cannabinoid is cannabidiol (CBD), which is non-intoxicating (Englund et al., 2017). CBD has a broad range of pharmacological actions, including inhibiting the reuptake and hydrolysis of anandamide as well as multiple receptor mechanisms (Bisogno et al., 2001; Laprairie et al., 2015). The receptor mechanisms include negative allosteric modulation of CB1 receptor, partial agonist or inverse agonist action on CB2 receptor, and agonist action on transient receptor potential vanilloid 1 (TRPV1) and serotonin 5-HT_1A_ receptor (Galaj and Xi, 2020), all of which are thought to lie behind CBD’s effects in reducing drug reward and addiction in preclinical studies (Navarrete et al., 2021).

Broadly speaking, cannabis products can be divided into four categories: high-THC-concentration/sinsemilla (unpollinated flower), herbal (pollinated flower, containing seeds and with lower THC), hash/resin (compressed cannabis trichomes along with plant matter), and very high-THC cannabis concentrates (such as hash oil, wax dabs, butane hash oil, shatter, etc.) (Potter et al., 2018). In the past two decades, dramatic increases in THC concentration in cannabis products have been reported across Europe (Dujourdy and Besacier, 2017; Freeman et al., 2020a, b; Potter et al., 2018; Rømer Thomsen et al., 2019) and the U.S. (ElSohly et al., 2016; Smart et al., 2017). For example, a large study from the U.S. found a general increase in THC concentration in seized cannabis plant material from about 4% in 1995 to about 12 % in 2014 (ElSohly et al., 2016), and a comprehensive study from France found a dramatic increase in THC concentration in seized cannabis resin from 10 % in 2009 to 23 % in 2016 (Dujourdy and Besacier, 2017). In line with these findings, a recent study found an alarming 3-fold increase in THC concentration in seized cannabis resin in Denmark from 8% in 2000 to 25 % in 2017 (Rømer Thomsen et al., 2019), which represents the highest concentration throughout Europe (Freeman et al., 2019b). During the same period several studies indicate that CBD concentrations have remained stable or even decreased; in the studies from France and Denmark, CBD concentration remained stable at around 4% and 6% (Dujourdy and Besacier, 2017; Rømer Thomsen et al., 2019). This development is highly concerning, as emerging studies show that cannabis products with high THC levels and low CBD levels may have more detrimental effects on cognitive function and mental health among both young and adult users (Englund et al., 2017; Rømer Thomsen et al., 2017; Wilson et al., 2019), including higher risk of cannabis-induced psychosis (Di Forti et al., 2019; Hjorthøj et al., 2019), impairments in learning and memory (D’Souza et al., 2004; Morgan et al., 2012; Morrison et al., 2009), and poorer addiction outcomes (Curran et al., 2018; Freeman and Winstock, 2015; Meier, 2017). Increasing THC levels have also been linked with increases in admission to CUD treatment in Europe (Freeman et al., 2018).

## 2. Cannabis use and adverse outcomes among youth

Cannabis use is most prevalent in adolescents and emerging adults. For example, 18 % of youth aged 15–24 in Europe report using cannabis the past year compared to 7 % of 25–64 year old’s (European Monitoring Centre for Drugs and Drug Addiction, 2019). In the U.S., about 25 % of adolescents report cannabis use by age 14, and recently rates of cannabis use have started to approximate adolescent alcohol use patterns with half of adolescents now using cannabis by age 18 (CDC, 2016; Johnston et al., 2018). Notably, the percentage of lifetime cannabis using adolescents who report frequent use (i.e., 20+ past-month occasions) are at the highest level in recent decades (14 %), reflecting a three-fold increase from the lowest reported levels in 1990–1991 (5.4 %) (Terry-McElrath et al., 2020).

Rates of CUD peak during adolescence and emerging adulthood, and the global burden of disease related to cannabis use peaks in emerging adulthood (ages 20–24) (Degenhardt et al., 2013). Individuals who initiate cannabis use during adolescence experience more adverse and long-lasting cannabis-related harms (Silvers et al., 2019; Volkow et al., 2014). About 30 % transition into CUD (Hasin et al., 2015; Volkow et al., 2014), and there is an increased risk of other substance use disorders (SUD) later in life (Grant et al., 2010). Particularly among youth, frequent cannabis use is linked with a wide range of mental health disorders, health risks, and poor psychosocial outcomes, including low life satisfaction, school dropout, lower educational and occupational achievements, behavioral and legal problems, accidents/injuries, sexually transmitted infections, and psychotic disorders during adulthood (Budney et al., 2007a; Coffey and Patton, 2016; Green et al., 2017, 2010; National Academies of Sciences, 2017).

The increased risk of adverse outcomes associated with frequent cannabis use among youth is related to the nature of the developing brain and the role of the endocannabinoid system in the neurodevelopmental maturation during adolescence (Wilson et al., 2019). The brain continues to develop from the prenatal period through childhood and adolescence until the beginning/mid 20 s (Gogtay et al., 2004). During these developmental periods, the brain is believed to be more vulnerable to the adverse effects of neurotoxins, such as regular exposure to cannabis. This means that the health impact and effects of cannabis, stemming from the increase in cannabis potency may pose unique risks for youth, because THC is likely to interfere with the neurodevelopmental processes influenced by the endocannabinoid system (Wilson et al., 2019). Related to this, a study of regular cannabis users and matched controls (Zalesky et al., 2012), found that cannabis use was associated with impaired axonal connectivity in the fimbria of the hippocampus (involved in learning and memory) and the precuneus (involved in self-awareness), and that early age of onset of regular use was associated with more severe microstructural white matter alteration. Additionally, a multisite longitudinal study in Europe found that cannabis use was associated with accelerated age-related cortical thinning between the ages of 14 and 19, predominantly in prefrontal regions with a high density of CB1 receptors (Albaugh et al., 2021). Further, studies examining response inhibition have shown that adolescents engaged in cannabis use require more neural resources to perform at the same level as non-using adolescents (after 1 month abstinence) (Tapert et al., 2007), or perform worse (Behan et al., 2014). However, longitudinal studies are required to determine if these differences are directly related to THC exposure or whether they may (also) predispose individuals for developing CUD. In the only randomized, placebo-controlled study of cannabis administration in adolescents and adults to date (Mokrysz et al., 2016), adolescents experienced heightened impairment of response inhibition and wanting more cannabis, alongside blunted subjective effects and memory impairment compared to adults.

## 3. Special considerations with psychosocial interventions for youth cannabis use

Preventing and intervening in the neurocognitive and health sequelae of early or frequent cannabis use is complex, and involves physical, psychological, medical, and cultural considerations. With regard to youth cannabis use, it is important to first consider the ways in which adolescents and emerging adults are developmentally distinct from adults in ways that have historically posed challenges to substance use prevention and treatment interventions in general (Cousijn et al., 2018; Feldstein Ewing et al., 2016b; Silvers et al., 2019). One barrier to preventing the onset and escalation of adolescent cannabis use is that across cultural contexts, experimentation with substances such as alcohol and cannabis often falls in line with cultural expectations, and is perceived as socially acceptable by the youth (Grønkjær et al., 2011; Järvinen and Ravn, 2014; Shedler and Block, 1990). However, the cultural and social context that influence an early onset of cannabis use may also have adverse neurocognitive implications for later brain and behavior function and ability (Lisdahl et al., 2013).

As summarized in Silvers et al. (2019), adolescence and emerging adulthood is a period with unique neurodevelopmental tasks that underscore major contrasts from adulthood: myriad pubertal changes, rapidly developing cognitive skills, an emergence of self-identity, and prominent changes in the social landscape. Accordingly, adolescent cognitive skills are also more directly tied and embedded in, and thus strongly impacted by, the socioemotional context and the broader ability to engage in self-regulatory cognitive strategies (e.g., reappraisal, emotional distancing) is only emerging and is markedly different from adults (Silvers et al., 2012). Because the ability for abstract reasoning and the capacity for purposeful and planful behavior is also in a developmental neurocognitive phase, this may subsequently impact the ability to participate meaningfully in established empirically supported behavioral treatment paradigms for substance use that have historically been designed for adults and largely validated with adult samples (Feldstein Ewing et al., 2016a). Exploring personal identity and a heightened sense of self also demarcates and differentiates the neurodevelopmental period of adolescence and emerging adulthood, and the formation of self-identity is itself contingent upon increased self-awareness and self-monitoring (Pfeifer and Berkman, 2018). Unique to this developmental period, enhanced self-focus and social attunement with the environment may motivate youth to use substances to improve social standing, particularly within peer contexts (Caouette and Feldstein Ewing, 2017; Cousijn et al., 2018). In contrast to adults, adolescents may also be more likely to take risks in the presence of their peers such that risk taking in social contexts heightens the potential reward value (driving task fMRI study; (Chein et al., 2011)). Of note, this penchant toward enhanced risk taking has now not only been examined as a potential risk factor, but also as a likely resilience factor, as adolescents also have an enhanced capacity to engage in prosocial “risks” when in peer contexts (Andrews et al., 2021; van de Groep et al., 2020). Peer influence is thus a powerful motivator for both risk taking and prosocial behaviors, highlighting the significance of addressing peer interactions in adolescent prevention and treatment interventions. These central facets of neurodevelopment in adolescence and emerging adulthood highlight key contextual distinctions between youth and adult cannabis users; the assumption that adult substance prevention and treatment models work equally well for youth is itself a barrier to developing novel approaches.

Another major challenge in addressing the significant public health issue that cannabis use presents for youth is that most youth who engage in frequent cannabis use do not seek or receive prevention and treatment interventions (Kerridge et al., 2017; Pedersen and Frederiksen, 2012; Silvers et al., 2019), even after years of harmful use and related negative health sequalae; on average, cannabis users have more than 10 years of near-daily use and more than 6 attempts at quitting, prior to seeking and/or receiving treatment (Budney, 2006). However, despite the high clinical importance, research on barriers to prevention and treatment interventions among youth with frequent cannabis use is surprisingly scarce, although some studies suggest that central barriers for seeking timely help involve social stigma, an overall desire to be self-reliant, believing intervention is not needed, or presuming that programs will be ineffective (Corrigan, 2004; Johnson et al., 2001; van der Pol et al., 2013). Additional complicating factors involve the increasing ease of access to cannabis, particularly in regions with permissive cannabis legislation (e.g., recreational cannabis laws), which has been directly tied to increases in youth cannabis use (Stormshak et al., 2019), perceiving cannabis use as harmless (Demant and Ravn, 2010), and increasing social acceptance of cannabis use among peers (Järvinen and Ravn, 2014; Søgaard et al., 2016). Moreover, positive associations with cannabis (Roditis and Halpern-Felsher, 2015) coupled with limited experience of, and anticipation for, negative consequences among adolescents and emerging adults, mean that this age group might not have a sensation of urgency to seek intervention either on the personal side (e.g., do not often wrestle with the feelings of ambivalence that often motivate adult substance users to seek treatment (Feldstein Ewing et al., 2016a) or on the social side (e.g. do not feel a pressure by their peer group to change and/or modify cannabis use behavior (Cousijn et al., 2018)). Related, once successfully engaged in prevention and treatment interventions for CUD, a significant challenge is retaining youth in the intervention (Lees et al., 2021). Further, the recent National Academies on Cannabis report (2017) also underscored the additional challenge of potential increase in other substance use (e.g., alcohol, vaping) (National Academies of Sciences et al., 2017). Taken together, to reach and help youth with/at-risk of developing CUD at an earlier stage, there is a dire need to develop and test new prevention and treatment interventions that are articulated specifically for this important neurodevelopmental population.

## 4. Meeting the challenges in cannabis using youth: psychosocial interventions

Psychosocial interventions for cannabis use can be employed at different stages prior to and into the development of CUD and have been assessed both in non-treatment seeking youth and in youth enrolled in treatment. In particular, an important avenue for circumventing treatment barriers and reaching non-treatment seekers, and maximizing prevention and treatment impact, is via brief, early evidence-based interventions that are non-judgmental and affirming (Feldstein Ewing et al., 2021, 2016a; Silvers et al., 2019). Motivational interviewing (MI; encompassing motivationally-based interventions including motivational enhancement therapy) is very well-suited to this end, because it is, by definition, non-judgmental, strength-based, affirming, empathic, and bolsters motivation for change (Jensen et al., 2011), and has shown to be highly acceptable and feasible among young cannabis users (Feldstein Ewing et al., 2013; Jensen et al., 2011; Martin et al., 2005; Stern et al., 2007). Prior studies have found that 2 sessions of MI can successfully reach and engage non-treatment-seeking young cannabis users and reduce cannabis use and related symptoms (Dupont et al., 2016; Martin and Copeland, 2008; McCambridge and Strang, 2004; Walker et al., 2016). However, compared to adults, effects of MI have generally been less impactful among youth (Hettema et al., 2005; Jensen et al., 2011). Combining brief, evidence-based psychological interventions like MI with safe and effective pharmacological treatment may represent an important avenue for reaching and engaging non-treatment seeking youth, helping them reduce or quit cannabis use, and facilitate a healthier trajectory.

Multiple psychosocial interventions have been investigated for adolescents and emerging adults enrolled in CUD treatment, however these interventions have typically been developed for use in adult (and not youth) populations and not for cannabis as the primary problem drug (Schettino et al., 2015; Silvers et al., 2019). Several reviews provide in-depth details on the nature and efficacy of each intervention approach (Gayes and Steele, 2014; Jensen et al., 2011; Steele et al., 2020; Winters et al., 2021), but examples include that the combination of MI and cognitive behavioral therapy (CBT) is associated with reduced cannabis use in adolescents with CUD. Typically, MI is employed over one or two sessions initially, in order to increase motivation to stop using cannabis and enhance ambivalence towards reducing use. This is followed by several sessions of CBT, with the aim of introducing coping skills that can be used in real-life settings and include devising plans for specific high-risk situations and developing problem solving skills. Recent studies have also shown that adding vouchers or monetary incentives for not using cannabis (contingency management) to weekly MI + CBT in youth has been associated with increased abstinence from cannabis (Stanger et al., 2015) and increased treatment retention (Pedersen et al., 2021) compared to weekly MI + CBT alone. Finally, some studies on multidimensional family therapy (MDFT), an approach that involves both the adolescent and the family member(s), have found that MDFT has comparable efficacy to CBT over 5–6 months of treatment, with evidence of increased treatment acceptability (Hendriks et al., 2011), and that a 6-month programme has been associated with good retention as well as reduced cannabis dependence compared to individual psychotherapy (Rigter et al., 2013). However, although these interventions have been associated with positive CUD outcomes, sustained abstinence is still only achieved in a minority of youth with CUD and treatment response tends to wane at follow-ups (Gates et al., 2016). Furthermore, most psychosocial treatment interventions require multiple sessions over several weeks or months and require high motivation and patient stability. Combining brief evidence-based psychosocial treatment options with pharmacotherapy represents a potentially important avenue to reach young cannabis users and improve treatment outcomes, e.g., through a potential increase in short-term recovery, which may help increase motivation into longer-term psychosocial interventions.

## 5. Meeting the challenges in cannabis using youth: cannabinoids as a new treatment strategy

Despite great effort, there are no approved pharmacotherapies for CUD in youth or adults. A 2019 Cochrane review found that abstinence at the end of treatment was no more likely with active pharmacotherapy with selective serotonin reuptake inhibitor (SSRI) antidepressants, mixed action antidepressants, anticonvulsants and mood stabilisers, buspirone and N-acetylcysteine compared to placebo (Nielsen et al., 2019). Of note, one RCT among adolescents with CUD (n = 116) found that eight-week treatment with N-acetylcysteine doubled the odds of abstinence from cannabis during treatment compared to placebo (Gray et al., 2012). However, a subsequent larger RCT in adults with CUD (n = 302) did not find evidence for benefits of 12-week N-acetylcysteine treatment on abstinence from cannabis compared to placebo (Gray et al., 2017).

Of relevance, abstinence at the end of treatment may not be a developmentally-appropriate intervention goal in cannabis using youth (or adults), and recent expert consensus on clinical outcomes for CUD trials recommend that sustained abstinence should not be the primary outcome for all clinical trials of CUD (Loflin et al., 2020). Thus, taking a harm reduction approach in treatment of CUD may weigh towards future studies on how to best attract youth with a frequent cannabis use/CUD to treatment that does not require abstinence, but targets reduction in use and in adverse consequences related to use.

The growing research and recognition of medical cannabis indicate the potential utility of cannabinoid-based-medicines as a strategy in treatment of CUD. Although it may sound initially counterintuitive, *cannabinoids* are receiving growing global interest as a safe and tolerated option for individuals with CUD. Because the divide between recreational and medical use of a drug may often overlap (such as in the case of opioids) (Pacula et al., 2016; Schlag et al., 2021), the use of cannabis in CUD treatment makes it imperative to address the many questions in order to be able to minimize potential harms of medical cannabis use. Generally speaking, an increasing number of countries have recognized potential medicinal uses of cannabinoid-based products and cannabinoids across Europe (Abuhasira et al., 2018; European Monitoring Centre for Drugs and Drug Addiction, 2018; Kvamme et al., 2021), the U. S. (National Conference of State Legislatures, 2020), Canada (Fischer et al., 2015), and Australia (Castle et al., 2019), providing greater flexibility for prescribers and facilitating research which has been difficult to conduct due to restrictions laid down on cannabinoid medicinal products (Braithwaite et al., 2019; Nutt et al., 2013; Shover and Humphreys, 2019). For example, there has been an increase in synthetic cannabinoid formulations such as nabilone (THC) licensed for the treatment of chemotherapy-induced nausea and vomiting, as well as nabiximols (THC + CBD) for the treatment of spasticity and multiple sclerosis, and Epidiolex (CBD) for the treatment of severe treatment resistant epilepsy in children (Freeman et al., 2019c).

So far, all studies on use of cannabinoids in treatment of CUD have been conducted in adults, while studies in youth are lacking. In the next sections, we review evidence from studies examining the effects of cannabinoids on cannabis use pathology among adult non-treatment seekers and patients in treatment and discuss the relevance of these findings for youth. Studies were found by searching PubMed, Embase, and PsycInfo databases with the terms “cannabis use disorder” or “cannabis dependence” or “cannabis treatment” combined with “cannabinoid” or “tetrahydrocannabinol” or “THC” or “cannabidiol” or “CBD” or “fatty acid amide hydrolase inhibitor” or “FAAH inhibitor”; and by checking reference lists of existing studies and reviews up to June 1st 2021. We did not include qualitative studies and case studies.

### 5.1. THC based cannabinoid agonist treatment strategies

THC is a partial agonist at CB1 and CB2 receptors (Howlett et al., 2002). Partial agonist action at CB1 receptor is responsible for the psychotropic effects associated with cannabis use as well as potentially therapeutic effects such as pain- and appetite-modulating actions. These clinical effects are mediated through CB1 receptor activation via inhibition of nociception, activation of reward pathways and regulation of mood, memory, and cognition (Zagzoog et al., 2020). Parallel effects of CB2 receptor activation include inhibition of the inflammatory response in cells of the immune system (Howlett et al., 2002). A number of studies have examined the effect of dronabinol or nabilone (synthetic THC) or nabiximols (THC and CBD at 1:1 ratio) on CUD symptoms in adults (Table 1). In general, THC based medicines are aimed at ameliorating cannabis withdrawal symptoms and craving when patients with CUD cease or reduce their use of illicit cannabis, because such compounds directly substitute the cannabis THC content. This treatment principle is similar to opioid and nicotine replacement therapy and is thought to provide a safer alternative that better facilitates participation in psychosocial and other treatment interventions (Lintzeris et al., 2019). By this mechanism, THC based cannabinoid agonist medicines may help decrease the risk of relapse due to withdrawal symptoms (Werneck et al., 2018).

Several laboratory studies have shown that dronabinol can reduce withdrawal symptoms in adult non-treatment seeking daily cannabis users (cannabis withdrawal symptoms typically include irritability, aggression, anxiety, insomnia, weight loss, restlessness, depressed mood, abdominal pain, tremors, sweating, and headache (Hesse and Thylstrup, 2013; Nielsen et al., 2020)). In an inpatient setting (N = 11 enrolled, 7 completed) (Haney et al., 2004), 10 mg of dronabinol 5 times/day decreased withdrawal symptoms and craving and produced no intoxication, compared to placebo. In an outpatient setting (N = 22 enrolled, 8 completed) (Budney et al., 2007b), dronabinol (10 mg or 30 mg 3 times/day) dose-dependently decreased withdrawal symptoms compared to placebo, with greater reductions following the high dose (30 mg 3 times/day). However, the high dose produced symptoms of cannabis intoxication and drug effects (euphoria and liking) compared to placebo. Vandrey et al. (2013) replicated the ability of dronabinol to dose-dependently decrease withdrawal symptoms (30, 60, or 120 mg/day) in adult, daily cannabis users (N = 25 enrolled, 13 completed). There was a significant effect of the high dose on dry mouth, rapid heart rate and flushing, and decrements in a minority of the cognitive performance measures. There is also some evidence that dronabinol (60 mg/day) combined with an alpha2-adrenergic receptor agonist, lofexidine (2.4 mg/day), produces synergistic effects for decreasing withdrawal symptoms, craving and relapse in adult non-treatment seeking daily cannabis users (N = 8 enrolled, completed not reported) (Haney et al., 2008).

A randomized controlled trial (RCT) (Levin et al., 2011) with cannabis dependent adults (N = 156 randomized, 99 completed) compared dronabinol (40 mg/day) with placebo over 12 weeks with concomitant weekly MI. Compared to placebo, dronabinol improved treatment retention and reduced withdrawal symptoms, but not cannabis use and 2-week abstinence. An RCT (Levin et al., 2016) with cannabis dependent adults (N = 122 randomized, 67 completed) compared a higher dose of concurrent dronabinol (60 mg/day) and lofexidine (1.8 mg/day) with placebo over 11 weeks with concomitant weekly MI, and found no effect of drug treatment on 3-week abstinence, withdrawal symptoms, or treatment retention.

A laboratory study of nabilone (Haney et al., 2013), which has a higher bioavailability than dronabinol, found that compared to placebo, 6 and 8 mg nabilone/day decreased withdrawal symptoms and relapse behavior in adult non-treatment seeking daily cannabis users (N = 12 enrolled, 11 completed). Nabilone did not increase ratings of “liking” or “desire to take again” compared to placebo, but 8 mg/day worsened cognitive task performance. A pilot RCT (Hill et al., 2017) comparing placebo and nabilone (2 mg/day) over 10 weeks in adults with cannabis dependence (N = 18 randomized, 12 completed), found that nabilone was safe and tolerated, but had no effect on cannabis use compared to placebo.

So far, findings from studies examining nabiximols’ (THC and CBD) efficacy on cannabis use pathology in adults are more mixed. A proof of concept study (Trigo et al., 2016) of fixed and self-titrated nabiximols use (up to 108 mg THC and 100 mg CBD) in non-treatment seeking cannabis dependent adults (N = 16 randomized, 9 completed), showed that high fixed doses were well tolerated and reduced withdrawal symptoms, but not craving, compared to placebo (Trigo et al., 2016). Self-titrated doses were lower and had limited efficacy compared to high fixed doses. A two-site inpatient RCT in treatment-seeking adult patients with CUD (N = 51 randomized, 19 completed) receiving 6 days of nabiximols (max 86.4 mg THC and 80 mg CBD/day) or placebo with concomitant MI/CBT during a 9-day admission, found that nabiximols reduced withdrawal and improved treatment retention (by day 6, not by day 9), but did not reduce cannabis use, dependence or cannabis-related problems compared to placebo (Allsop et al., 2014). Nabiximols was not associated with greater intoxication or adverse events. A pilot, outpatient RCT (Trigo et al., 2018) in treatment seeking cannabis dependent adults (N = 40 randomized, 27 completed) receiving 12-week treatment with self-titrated nabiximols use (up to 113.4 mg THC and 105 mg CBD) or placebo, concurrent with weekly MI and CBT, found that nabiximols was well tolerated with no serious adverse events, but had no effect on withdrawal symptoms, cannabis use and abstinence, compared to placebo. In contrast, a multi-site, outpatient RCT (Lintzeris et al., 2019) in cannabis dependent adults (N = 137 randomized, 60 completed) receiving 12-week treatment with self-titrated nabiximols use (up to 86.4 mg THC and 80 mg CBD) or placebo, concurrent with 6 sessions of CBT, found that nabiximols was well tolerated with few adverse events, had no effect on withdrawal symptoms, craving and cannabis-related problems, but reduced cannabis use during the trial, compared with placebo. The reduction in cannabis use in the nabiximols group, compared to placebo, was maintained 12 weeks after treatment (Lintzeris et al., 2020).

To summarize, the evidence from placebo-controlled laboratory studies suggests that treatment with dronabinol (ranging from 30 to 120 mg/day) dose-dependently decrease withdrawal symptoms, with some evidence that higher doses produce cannabis intoxication and drug liking. Evidence from large placebo-controlled RCTs in cannabis dependent adults (N > 100 enrolled) combining dronabinol with MI over 11–12 weeks partly supports this: 40 mg/day reduced withdrawal symptoms and improved treatment retention, but not cannabis use and abstinence (Levin et al., 2011), but a higher dose, 60 mg/day combined with lofexidine (1.8 mg/day) had no effect on neither withdrawal symptoms nor abstinence or treatment retention (Levin et al., 2016). Nabilone has been less examined, but the evidence from placebo-controlled studies is similar: a laboratory study suggests that 6 or 8 mg/day reduces withdrawal symptoms (without increasing drug liking), and a small RCT (2 mg nabilone/day over 10 weeks) in cannabis dependent adults found no effect on cannabis use. Evidence from placebo-controlled studies of nabiximols combined with MI and CBT is more mixed: out of the three conducted RCTs in cannabis dependent adults, self-titrated nabiximols reduced withdrawal symptoms, but had no effect on cannabis use, dependence or cannabis-related problems in one study (max 86.4 mg THC and 80 mg CBD/day during 9-day admission) (Allsop et al., 2014); had no effect on neither withdrawal symptoms nor cannabis use or abstinence in another study with a higher dose (max 113.4 mg THC and 105 mg CBD during 12 weeks) (Trigo et al., 2018); and had no effect on withdrawal symptoms, craving and cannabis-realted problems, but reduced cannabis use in a large study (N > 100; max 86.4 mg THC and 80 mg CBD during 12 weeks) (Lintzeris et al., 2019).

### 5.2. Non-THC based cannabinoid treatment strategies

Recently, studies have also examined the effect of FAAH inhibitors or CBD in adults with CUD (Table 1).

FAAH forms a part of the endocannabinoid system by breaking down endocannabinoids such as anandamide. By inhibiting this breakdown process, FAAH inhibitors increase endocannabinoid levels, which may represent a therapeutic mechanism for the treatment of CUD. Preclinical research has shown that FAAH inhibitors can attenuate cannabis withdrawal symptoms in THC-dependent mice (Schlosburg et al., 2009). So far, only one RCT has examined the effects of FAAH inhibitor treatment on cannabis use and cannabis pathology in humans (D’Souza et al., 2019). In a double-blind, place-controlled, parallel group phase 2a trial in adults with cannabis dependence (N = 70 randomized, 58 completed), participants received 4 mg daily PF-04457845 (N = 46) or placebo (N = 24) during 4 weeks. Relative to placebo, treatment with PF-04457845 reduced symptoms of cannabis withdrawal, and self-reported cannabis use at 4 weeks, which was confirmed by lower urinary THC-COOH concentrations. Further, there was no difference between reported adverse events and retention in the two groups.

A novel strategy with promising findings is also underway in preclinical and clinical studies of CBD (Batalla et al., 2019; Bonaccorso et al., 2019; Russo, 2011). For example, preclinical studies show that CBD attenuates drug-seeking behavior (Bonaccorso et al., 2019; Leaver et al., 2009), and a study found that CBD reversed the reinforcing effects of cannabis in youth (Morgan et al., 2010), supporting CUD treatment potential. The multiple receptor mechanisms of CBD outlined earlier are believed to form the neurobiological underpinnings of the effects of CBD on the regulation of reinforcing, motivational and withdrawal-related effects as documented in preclinical and clinical studies (Navarrete et al., 2021). It is still not known precisely how CBD interacts with the dopaminergic system to modify the motivational effects of psychoactive drugs, but available data suggest a role for CBD in regulating the activity of the mesolimbic dopaminergic system (Navarrete et al., 2021). This role has been emphasized by the localization of cannabinoid receptors in the mesolimbic circuit orchestrating the synthesis and release of dopamine (Manzanares et al., 2018). Apart from the interaction with the endocannabinoid system through alteration of endocannabinoid signaling, the effect of CBD on drug addiction has been shown to involve modest affinity agonist action at 5-HT_1A_ receptors (Russo et al., 2005).

So far, two studies have examined the effects of CBD in adult cannabis users (Freeman et al., 2020b; Solowij et al., 2018). A pragmatic open-label clinical trial evaluated the effect of 10 weeks of 200 mg daily CBD administration on psychological symptoms and cognition in adults with frequent cannabis use (N = 20 enrolled), while participants continued cannabis use (Solowij et al., 2018). Compared with baseline, participants reported fewer depressive and psychotic symptoms, but more state anxiety symptoms after CBD treatment, and demonstrated improvement in attentional switching, verbal learning, and memory. CBD was well tolerated with no reported side effects, in line with a recent meta-analysis of CBD clinical trials reflecting that CBD tends to be very well-tolerated with few serious adverse effects (e.g. diarrhea) (Chesney et al., 2020). Participants retrospectively reported reduced euphoria when smoking cannabis, in line with previous studies showing that CBD reverses the reinforcing effects of cannabis in youth (Morgan et al., 2010). The study provided the first evidence that prolonged CBD treatment may improve psychological symptoms and cognitive function in frequent users, with greater benefits in dependent than in nondependent cannabis users. However, the study lacked a placebo control and was not designed to examine effects on cannabis use.

Recently, Freeman and colleagues (Freeman et al., 2020b) offered the first empirical data supporting that a treatment package comprised of CBD + MI was able to engage non-treatment seeking adults with CUD and reduce post-treatment cannabis use. In a phase 2a dose-finding RCT, they found that synthetic CBD over a 4-week treatment period outperformed placebo (N = 23) at daily doses of 400 mg (N = 24) or 800 mg (N = 23) (but not 200 mg (N = 12); eliminated at interim analysis due to inefficacy) in reducing biological and self-report metrics of cannabis use among a sample of non-treatment seeking adults with CUD, who wanted to quit cannabis use (N = 82 randomized, 77 completed) (Freeman et al., 2020b). Reductions in use were maintained up to 24 weeks following treatment with 400 mg CBD. Results on secondary outcomes were mixed. However, there was some evidence for CBD reducing cigarette use, cannabis withdrawal symptoms, and anxiety. By contrast, there was no evidence for changes in alcohol use, and there was evidence for a decrease in sleep quality following CBD treatment. Importantly, in terms of potential disruption in functioning, use of CBD was not associated with more adverse events than placebo – in line with the open-label trial (Solowij et al., 2018). Furthermore, the study showed impressive retention rates with 94 % retention throughout the study period.

To summarize, very few studies have examined the effect of non-THC based cannabinoid treatment strategies on CUD to date, but evidence from the first studies is promising. In the first placebo-controlled RCT in adults with cannabis dependence, FAAH inhibitors (4 mg/day over 4 weeks) reduced withdrawal symptoms and cannabis use compared to placebo, with no difference in adverse outcomes between groups. A pragmatic open label trial of CBD (200 mg/day over 10 weeks) in adults who continued their frequent cannabis use found improvements in depressive and psychotic symptoms, attention, learning and memory, and reduced euphoria when smoking cannabis. In the first placebo-controlled RCT of CBD in adults with cannabis dependence, CBD (400 or 800 mg/day over 4 weeks concomitant with MI) reduced cannabis use, with no difference in adverse outcomes between groups. Larger trials are needed to provide more precise estimates of efficacy for these novel treatment strategies.

### 5.3. Relevance of cannabinoid treatment for youth

As reviewed in Section 5.1, evidence from randomized placebo-controlled studies suggests that treatment with synthetic THC (dronabinol, nabilone) decreases withdrawal symptoms, but not cannabis use, in adults with daily cannabis use or CUD. Further, the same studies suggest that higher doses of THC may increase cannabis intoxication and liking, while the evidence regarding THC combined with CBD (nabiximols) is more mixed. The lack of effect on cannabis use and abstinence in adult studies with synthetic THC conducted so far, including studies where THC is administered concomitantly with MI/CBT up to 12 weeks, suggests that the efficacy of synthetic THC as a treatment among youth with CUD should not be tested as a stand-alone pharmacological treatment. The relatively consistent effect on withdrawal symptoms may suggest potential for combining dronabinol or nabilone with other medications, however, this should be weighed against potential negative outcomes associated with administering THC in youth samples.

More specifically, the evidence from laboratory studies suggests that higher doses of THC may increase cannabis intoxication and drug liking, which naturally raises concern, as these effects may pose a risk for relapse in patients who have stopped using cannabis and may be even more problematic in patients that aim to reduce, but not stop, using cannabis. In general, administering THC as treatment for CUD carries an element of clinical and ethical concern due to the emerging evidence suggesting that cannabis products with high levels of THC have detrimental effects on mental health and cognitive function among adult and youth users (Englund et al., 2017; Rømer Thomsen et al., 2017; Wilson et al., 2019). These concerns are very relevant considering the high rates of psychiatric co-morbidity and negative neurocognitive impact in youth and adults with CUD (Hasin, 2018; Volkow et al., 2014). Furthermore, the concerns are particularly pertinent with adolescents and emerging adults, as the developing brain is believed to be more susceptible to the adverse effects of cannabis exposure, and THC may interfere with neurodevelopmental processes influenced by the endocannabinoid system (Wilson et al., 2019). The majority of studies reviewed in Section 5.1 did not examine potential effects on cognitive function. However, in the studies that included cognitive assessment, findings suggest that THC-containing cannabinoid treatment decrease cognitive performance: one study found that 8 mg/day of nabilone (but not 6 mg/day) worsened psychomotor task performance (Haney et al., 2013), and another found that dronabinol worsened psychomotor task performance and working memory (Vandrey et al., 2013). While studies of nabiximols reported no differences in adverse events between treatment and placebo groups (Allsop et al., 2011; Lintzeris et al., 2019; Trigo et al., 2016, 2018), some studies found adverse events related to dronabinol, such as dry mouth, rapid hear, flushing and hypotension (Levin et al., 2016; Vandrey et al., 2013). In studies that reported serious adverse events, these were deemed not study related (Levin et al., 2011, 2016). A final point that is relevant to consider is that THC may interfere with daily functioning, because of its cognitive impairing effects. For example, dronabinol has been found to impair driving performance in a dose-dependent manner (Bosker et al., 2012), which has implications for potential risk of road accidents attributable to medicinal use of THC in treatment of CUD.

As reviewed in Section 5.2, the effect of FAAH inhibitors and CBD on CUD has been much less studied, but evidence from the first two placebo-controlled RCTs in adults with cannabis dependence suggests that FAAH inhibitors can reduce withdrawal symptoms and cannabis use, and that CBD can reduce cannabis use. Furthermore, there were no differences between control groups and groups receiving FAAH inhibitors or CBD regarding adverse outcomes (D’Souza et al., 2019; Freeman et al., 2020b; Solowij et al., 2018).

A key concern about administering pharmacotherapies to youth is their safety. In the only trial to date, the FAAH inhibitor PF-04457845 was administered to male adults aged 18–55. The trial did not include females of childbearing potential due to the previous lack of safety and toxicity data on PF-04457845. Now that these data are available, a subsequent phase 2b trial is being conducted in males and females aged 18–60 (D’Souza, 2021). Due to a lack of safety or toxicity data in younger people, the potential of PF-04457845 in treating youth CUD is currently unclear. There is also limited data on the safety of THC administration in youth, with only one study to date administering THC to adolescent volunteers (Mokrysz et al., 2016), as well as the already mentioned concerns about the impact on the developing brain. While the safety and efficacy of CBD for treating CUD in youth is yet to be established, the potential safety of administering CBD is supported by several trials of CBD as a treatment for severe treatment-resistant epilepsy in children (Devinsky et al., 2017, 2018; Thiele et al., 2018). Furthermore, an emerging literature suggests that CBD contains opposing neural, cognitive, and behavioral effects that interact with, and may counteract, some of the harmful effects of THC on cognitive functions, anxiety, and psychotic symptoms (Blessing et al., 2015; Bonaccorso et al., 2019; Englund et al., 2017; Freeman et al., 2019a; Gunasekera et al., 2020; Iseger and Bossong, 2015; Osborne et al., 2017; Rømer Thomsen et al., 2017). Some studies also point to a greater addiction potential in products with high levels of THC and low levels of CBD (Curran et al., 2018; Freeman and Winstock, 2015; Meier, 2017; Morgan et al., 2010). These effects are mirrored in functional imaging studies, which have revealed opposing acute effects of THC and CBD in areas pivotal to the examined cognitive processes including amygdala (processing of emotional information); striatum, hippocampus, and prefrontal cortex (processing of salience); and auditory and visual cortex (processing of auditory and visual information (Bhattacharyya et al., 2010; Borgwardt et al., 2008; Fusar-Poli et al., 2009; Winton-Brown et al., 2011). Taken together, these findings point to a safer profile of CBD compared to THC.

A potential benefit of cannabinoid treatment in general is that it may be more acceptable than traditional treatment (and other medications) among cannabis using youth and adults, especially non-treatment seekers. In the only placebo-controlled RCT conducted with CBD so far, retention rates were very high: out of 82 randomized individuals with CUD, only 5 participants did not complete treatment (Freeman et al., 2020b), and in the only placebo-controlled RCT of FAAH inhibitors, 17 % dropped out of placebo and active treatment groups (D’Souza et al., 2019). Overall, drop-out rates were higher and more mixed in studies on THC-based cannabinoid treatment, see also Table 1. As such, cannabinoid medication – particularly non-THC based - may potentially help address the problem of low treatment uptake in youth.

## 6. Recommendations for future studies

This is an extremely exciting time in the domain of adolescent and emerging adult prevention and intervention work for cannabis use and related CUD. At present, we are coming out from an era with limited prevention and treatment intervention options. To that end, the past few decades have been demarcated by addiction paradigms and related prevention and intervention approaches that were solely developed for and examined with adults. On that background, it is highly promising that the field is increasingly recognizing adolescence and emerging adulthood as a distinct neurodevelopmental period with its own unique needs and challenges that are also mirrored in the needs and challenges related to cannabis use (Feldstein Ewing et al., 2018; Giedd, 2015).

This review represents a comprehensive overview of how exciting advances in the domain of emergent cannabinoid treatment strategies may open a critical and important window for the abatement of youths’ struggles with cannabis. With the increase in cannabis use observed throughout the pandemic (Cousijn et al., 2021), these steps could not be more timely.

Examinations of cannabinoids as treatment strategies for CUD have so far been restricted to adult populations. These studies have yielded promising findings regarding FAAH inhibitors and CBD, but large efficacy trials are still needed to replicate the first findings. The optimal duration of treatment with cannabinoid substitution treatment has also not yet been established and needs to be systematically investigated together with risk of relapse after cannabinoid treatment has been discontinued. This is of particular importance, because of the high price of some of the cannabinoid agonist compounds recently released to the market.

With no studies to date having examined the potential of cannabinoid treatment in youth with CUD, the next critical steps include examining the impact of these novel intervention approaches (e.g., CBD+/− psychosocial approaches) in youth samples, in order to examine potential effects and side effects, and determine which elements in the approach are driving specific changes in youth behavior and brain. To this end, translational approaches integrating examinations with behavioral, neurocognitive, and neuroimaging (MRI/fMRI) can be particularly useful in disaggregating potential impact and neural- as well as behavioral- mechanisms of change in this important population (Ray and Grodin, 2021; Ray et al., 2021). Furthermore, examinations of potential gender differences are needed - both in youth and adult samples. Fewer females use cannabis, but cannabis use is linked to more health problems in young females compared to males (Girgis et al., 2020; Hesse et al., 2020). However, research in females and potential gender differences is lacking in cannabis research in general (Del Boca, 2016; Hamilton and Monaghan, 2019; Lees et al., 2021), including research on cannabinoid treatment, where previous studies have either examined 100 % males or mainly males. So far, no studies have examined potential gender differences in effects of cannabinoid treatment (see Table 1).

Together, this work represents critical next steps in the important pathway toward improving the lives of young people struggling with CUD. In the longer run, once tolerability and safety of cannabinoid treatment strategies have been established in youth, more specific target groups and treatment strategies could be further explored. For example, the therapeutic effects of pharmacological treatment with CBD (i.e., antipsychotic, anxiolytic) have the potential to guide the definition of CUD subgroups that might in particular benefit from this treatment strategy. Since THC-based compounds seem rather efficient in reducing withdrawal symptoms, another approach might be to examine the efficacy of administering both THC and CBD in the beginning of treatment and then gradually phase out to end with CBD monotherapy. However, due to the discussed risks and concerns associated with THC administration, this approach requires careful ethical and clinical considerations.

## Figures and Tables

**Table 1:** Studies examining cannabinoids as a treatment strategy for CUD.

Study	Cannabinoid treatment	Sample and Design	Findings: Primary outcomes	Adverse events	Retention in trial
**THC based cannabinoid agonist treatment strategies**					
(Haney et al., 2004)	10 mg dronabinol 5 times/day, or placebo. Two 15-day in-patient phases, counterbalanced and separated by 5-day outpatient period.	11 adult regular cannabis users enrolled (100 % male), 7 completed.Age, Mean (SD): 24 (1). Within-subjects, crossover design. Age, Mean (SD): 24 (1). Within-subjects, crossover design.	Dronabinol decreased withdrawal symptoms compared to placebo during cannabis abstinence. Dronabinol decreased ratings of cannabis craving compared to placebo. Dronabinol produced no intoxication.	One case of stomach discomfort during cannabis withdrawal, and one case of abnormal ECG during cannabis withdrawal; both in the placebo condition.	4 non-completers (37 %).
(Budney et al., 2007b)	10 mg, 30 mg dronabinol, or placebo, administered 3 times/day for 5 days of cannabis abstinence, separated by 7–9-day smoking as usual periods.	22 adult, daily cannabis users enrolled, 8 completed (75 % male). Age, Mean: 32.5.Within-subjects, crossover design. Within-subjects, crossover design.	Dose-dependent decrease in withdrawal symptoms. The high dose produced symptoms of cannabis intoxication and drug effects.	No serious adverse effects, signs of cannabis-like intoxication in two participants in 30 mg condition, one report of severe strange dream during 30 mg condition.	14 non-completers (63 %).
Vandrey et al. (2013)	30 mg/day, 60 mg/day, 120 mg/day dronabinol, or placebo, for four 5-day inpatient periods, separated by 9-day washout periods.	25 adult daily cannabis users enrolled, 13 completed (92 % male). Age, Mean (SD): 34 (9). Within-subject crossover design.	Dose-dependent decrease in withdrawal symptoms.	Dry mouth, rapid heart rate, and flushing in 120 mg condition.Few adverse side effects or decrements in cognitive performance.No serious adverse events.	12 non-completers (48 %).
(Haney et al., 2008)	Dronabinol 60 mg/day + lofexidine 2.4 mg/day, or placebo, 7-day inpatient periods, separated by 8–11-day outpatient washout periods.	8 non-treatment seeking adult daily cannabis users (100 % male), completed not reported. Age, Mean (SD): 29 (7). Within-subject crossover design.	Dronabinol decreased some withdrawal symptoms but did not decrease relapse to cannabis use compared to placebo.Combined dronabinol and lofexidine decreased cannabis withdrawal, craving and relapse compared to either medication alone.	Not reported	Not reported
(Levin et al., 2011)	Dronabinol 40 mg/day (n = 79) or placebo (n = 77).12-weeks with concomitant weekly MI: 1-week placebo lead-in phase, 1-week medication titration phase, 6-week medication maintenance phase, 2-week dose taper phase, 2-week placebo lead-out phase.	156 adults with cannabis dependence randomized (82 % male), 99 completed. Age, Mean (SD): Treatment = 36.9 (10.8). Placebo = 38.4 (9.2). Double-blind, randomized control trial.	Dronabinol reduced withdrawal symptoms, but not cannabis use or 2-week abstinence compared to placebo.	No difference between reported adverse events in the two groups.Four serious adverse events, none deemed to be study-related.	33 non-completers in placebo group (43 %), 24 non-completers in dronabinol group (30 %).
(Levin et al., 2016)	Dronabinol 60 mg/day +1.8 mg/day lofexidine (n = 61) or placebo (n = 61).11-weeks with concomitant weekly MI, 1-week placebo lead-in phase, medications maintained until end of week 8 and then tapered.	122 adults with cannabis dependence randomized (69 % male), 67 completed. Age, Mean (SD): Treatment = 34.8 (11.2). Placebo = 35.4 (10.8). Double-blind, randomized control trial.	No effect of drug treatment on 3-week abstinence, withdrawal symptoms or treatment retention.	Dry mouth, intoxication and hypotension more common in treatment than placebo group.Two serious adverse events, neither deemed to be study-related.	26 non-completers in placebo group (43 %), 29 non-completers in Dronabinol + lofexidine group (48 %).
(Haney et al., 2013)	6 mg/day, 8 mg/day nabilone, or placebo, 8-day inpatient phases, separated by 7 outpatient washout days.	12 adult daily cannabis users enrolled, 11 completed (73 % male). Age, Mean (SD): 30 (10). Within-subject, crossover study.	6 and 8 mg nabilone/day decreased withdrawal symptoms and relapse behaviour.	8 mg nabilone significantly worsened cognitive task performance.6 and 8 mg nabilone doses lowered seated systolic pressure and diastolic pressure.	1 non-completer in placebo group (8%).
(Hill et al., 2017)	Nabilone 2 mg/day (n = 10) or placebo (n = 8).10 weeks with weekly behavioural intervention.	18 adults with cannabis dependence randomized (67 % male), 12 completed (75 % male). Age, Mean (SD): Treatment = 24.4 (5.2). Placebo = 28.9 (7.5). Randomized pilot study.	Nabilone did not reduce cannabis use compared to placebo.	8 reported adverse events rated mild-moderate.No serious adverse events reported.	4 non-completers in nabilone group (22 %), 2 non-completers in placebo group (11 %).
(Trigo et al., 2016)	Fixed and self-titrated nabiximols use (up to 108 mg THC and 100 mg CBD), fixed and self-titrated placebo. Four, 5-day smoke-as-usual conditions, separated by abstinence conditions.8-weeks, with concurrent MI + CBT.	16 cannabis dependent adults randomized, 9 completed treatment (89 % male). Age, Mean (SD): 35.9 (11.5). Double-blind, placebo-controlled, counterbalanced proof of concept trial.	Both nabiximols conditions reduced withdrawal compared to their corresponding placebo condition.Nabiximols conditions did not reduce craving scores compared to placebo conditions.	No difference between reported adverse events in the two groups.No serious adverse events reported.	7 non-completers (44 %).
(Allsop et al., 2014)	Nabiximols (max 86.4 mg THC and 80 mg CBD/day; n = 27) or placebo (n = 24)9-days, concomitant MI/CBT.	51 treatment-seeking adult patients with CUD randomized (76 % male), 19 completed. Age, Mean (SD): Treatment = 35.0 (9.7). Placebo = 35.0 (13.1). Two-site, double-blind, inpatient RCT.	Nabiximols reduced withdrawal and improved retention (by day 6, not day 9), but did not reduce cannabis use, dependence or cannabis-related problems compared to placebo.	Number and severity of adverse events did not differ between treatment and placebo	By day 9: 16 non-completers in nabiximols group (59 %), 16 non-completers in placebo group (67 %).
(Trigo et al., 2018)	Self-titrated nabiximols use (up to 113.4 mg THC and 105 mg CBD; n = 20).Or placebo (n = 20)12-week, concomitant weekly MI + CBT.	40 treatment seeking cannabis dependent adults randomized (73 % male), 27 completed. Age, Mean (SD): Treatment = 30.7 (10.4). Placebo = 35.3 (13.1). Double-blind, placebo-controlled outpatient RCT.	Nabiximols did not reduce withdrawal symptoms or cannabis use, or increase abstinence compared to placebo.	No difference between reported adverse events in the two groups.No serious adverse events.	7 non-completers in treatment group (20 %), 6 non-completers in placebo (30 %).
(Lintzeris et al., 2019)	Self-titrated nabiximols use (up to 86.4 mg THC and 80 mg CBD; n = 64) or placebo (n = 73).12-week, concurrent 6 sessions of CBT.	137 cannabis dependent adults randomized (77 % male), 60 completed. Age, Mean (SD): Treatment = 36.2 (11.5). Placebo = 33.8 (10.3). Multi-site, outpatient, parallel, double-blind phase 3 RCT.	Nabiximols had no effect on withdrawal symptoms, craving and cannabis-related problems compared to placebo.Nabiximols reduced cannabis use during the trial which was maintained 12 weeks after treatment (Lintzeris, 2020).	No difference between reported adverse events in the two groups.One serious adverse event in the placebo group.	34 non-completers in nabiximols group (47 %), 43 non-completers in placebo group (59 %).
**Non-THC based cannabinoid treatment strategies**					
(D’Souza et al., 2019)	FAAH Inhibitors: 4 mg daily PF-04457845 (n = 46) or placebo (n = 24) over 4 weeks:1-week at hospital to achieve abstinence, 3 weeks outpatient.	70 adults with cannabis dependence randomized (100 % male), 58 completed. Age, Mean (SD): Treatment: 28.5 (8.5). Placebo: 27.5 (8.7). Double-blind, place-controlled, parallel group phase 2a trial.	Relative to placebo, treatment with PF-04457845 reduced symptoms of cannabis withdrawal during hospital admission and self-reported cannabis use at 4 weeks, confirmed by lower urinary THC-COOH concentrations.	No difference between reported adverse events in the two groups.No serious adverse events.	4 non-completers in placebo group (17 %), 8 non-completers in PF-04457845 (17 %).
(Solowij et al., 2018)	200 mg CBD daily over 10 weeks.	20 adults (80 % male) with frequent cannabis use, who continued cannabis use. Age, Mean (Range): 25.1 (20.6–46.8). Pragmatic open-label clinical trial, without placebo-control.	Severity of depressive symptoms, psychotic-like symptoms and attentional switching, verbal learning and memory performance was improved at end of treatment compared to baseline.Anxiety was increased at the end of treatment compared to baseline.	No adverse events reported in the trial.	One participant completed only 5.5 weeks of treatment, four participants had 11–12 weeks of treatment.
(Freeman et al., 2020b)	Daily 200 mg (n = 12), 400 mg (n = 24) or 800 mg (n = 23) CBD, or placebo (n = 23).4-week treatment + concurrent MI.	82 adults with at least moderate CUD randomized (72 % male), 77 completed. Age, Mean (Range): 200 mg: 27.33 (19.28–39.08) 400 mg: 26.58 (19.15–41.25) 800 mg: 27.43 (19.00–36.90) Placebo: 24.87 (18.55–43.35) Double-blind, placebo-controlled, parallel group phase 2a trial.	400 mg and 800 mg CBD decreased urinary THC: COOH and increased days abstinent, compared to placebo.Reductions in use were maintained up to 24 weeks following treatment with 400 mg.	No difference between reported adverse events across treatment groups vs placebo.No serious adverse events.	2 non-completers in placebo group (9%), 2 in 200 mg CBD (17 %), 1 in 400 mg CBD (4%), and 0 in 800 mg CBD.
